# Simultaneously Modulating HIF-1α and HIF-2α and Optimizing Macrophage Polarization through the Biomimetic Gene Vector toward the Treatment of Osteoarthritis

**DOI:** 10.34133/bmr.0059

**Published:** 2024-07-29

**Authors:** Boyuan Zheng, Yiwan Shi, Lei Xiao, Bowei Li, Zihang Chen, Jing Zhao, Shaoping Li, Huige Hou, Jieruo Li, Xianlong Cai, Huajun Wang, Peng Wu, Xiaofei Zheng

**Affiliations:** ^1^ Department of Sports Medicine, The First Affiliated Hospital, Guangdong Provincial Key Laboratory of Speed Capability, The Guangzhou Key Laboratory of Precision Orthopedics and Regenerative Medicine, State Key Laboratory of Frigid Zone Cardiovascular Diseases, Jinan University, 510630 Guangzhou, China.; ^2^ Department of Orthopedics, Shanghai Tenth People’s Hospital, Tongji University School of Medicine, 200072 Shanghai, China.; ^3^ Department of Psychology, Li Ka Shing Faculty of Medicine, State Key Laboratory of Brain and Cognitive Sciences, The University of Hong Kong, Hong Kong SAR, China.; ^4^ Joint Laboratory of Chinese Herbal Glycoengineering and Testing Technology, University of Macau and National Glycoengineering Research Center, Macao, China.; ^5^ State Key Laboratory of Quality Research in Chinese Medicine, Institute of Chinese Medical Sciences, Department of Pharmaceutical Sciences, Faculty of Health Sciences, University of Macau, Macao, China.

## Abstract

In osteoarthritis (OA), articular cartilage is continuously submerged in a hypoxic environment throughout life, and hypoxia-inducible factors (HIFs) play a crucial role in OA progression. Among the various HIF phenotypes, HIF-1α positively contributes to maintaining the stability of the articular cartilage matrix. In contrast, HIF-2α has a detrimental effect, leading to chondrocyte apoptosis and exacerbating inflammation. Notably, there is currently no simultaneous regulation of HIF-1α and HIF-2α for OA treatment. Thus, the biomimetic gene vector (MENP) was developed for co-delivery of siHIF-2α and Mg^2+^ to the inflamed regions in OA joints, comprising an inner core consisting of siHIF-2α and Mg^2+^ and an outer M2 macrophage membrane. In vitro and in vivo studies demonstrate that MENP effectively targets inflamed areas, efficiently silences HIF-2α, and facilitates HIF-1α-mediated cartilage restoration through Mg^2+^. Furthermore, it indirectly promotes the polarization of macrophages toward an anti-inflammatory M2 phenotype through its action on inflamed synoviocytes. Overall, MENP is an efficient biomimetic vehicle for alleviating inflammation and promoting cartilage repair, representing an appealing approach for OA treatment.

## Introduction

Osteoarthritis (OA) is a prevalent age-related skeletal condition that affects the entire joint [1]. OA affects more than 300 million people worldwide, leading to chronic pain and disability [2,3]. A was previously attributed solely to articular cartilage wear and tear. However, it is now recognized as a chronic disorder affecting the entire joint, characterized by initial biochemical and cellular alterations within synovial joint tissues. These changes contribute to subsequent histological and structural modifications in the joint, ultimately resulting in overall tissue dysfunction. OA is thus understood as a complex, multifaceted condition involving pathological processes that extend beyond cartilage degradation to encompass broader joint pathology [4,5]. Articular cartilage, being an avascular connective tissue, is continuously submerged in a hypoxic environment throughout life [6–8]. Hypoxia-inducible factors (HIFs) mediate the response of chondrocytes to hypoxia during OA progression. HIF-1α supports metabolic adaptation to the hypoxic environment, maintaining the stability of articular cartilage, while HIF-2α induces apoptosis and amplifies the inflammatory response in chondrocytes, playing a regulatory role [9–11]. Taken together, simultaneous manipulation of HIF-1α and HIF-2α could represent a promising approach to the treatment of OA.

RNA interference is a highly precise and efficient therapeutic approach that offers a level of specificity not achievable by small-molecule inhibitors [12,13]. Small interfering RNA (siRNA) therapeutics utilizing nanocarriers hold great promise for the treatment of arthritic diseases [14–17]. Nanocarriers loaded with HIF-2α siRNA (siHIF-2α) can treat OA by inhibiting HIF-2α, preserving cartilage integrity, and reducing cartilage degeneration and synovitis [18]. The biomimetic vector, as a novel drug delivery system, can combine the advantages of synthetic carriers and incorporate multiple functions to address challenges in every step of RNA delivery [19]. The biomimetic carrier not only has excellent blood circulation and biocompatibility, but also is capable of acquiring diverse targeting properties through inherent features or intentional modifications, making it a promising siRNA delivery system [20–22]. Nanoparticles coated with macrophage membranes are highly efficient at targeting delivery and exhibit moderate therapeutic efficacy in inflammatory diseases such as rheumatoid arthritis, cancer, and sepsis [23–26]. Therefore, the macrophage membranes’ biomimetic siRNA carrier is expected to be suitable for the treatment of OA by down-regulating the expression of HIF-2α.

Recently, magnesium has been recognized as one of the most attractive biomaterials for research and application in orthopedics [27,28]. Mg-based biomaterials have excellent tissue compatibility and pro-osteogenic property, making them favorable for cartilage regeneration [29,30]. Moreover, a study has demonstrated that magnesium ions (Mg^2+^) facilitate chondrocyte proliferation and differentiation by up-regulating HIF-1α, which ultimately alleviates cartilage degradation in OA [31]. However, owing to the complex etiology of OA, the therapeutic effect of Mg^2+^ alone cannot be sustained for long periods of time, and will diminish within a relatively short period of time after cessation of treatment [32]. It is worth investigating whether the combination of HIF-2α siRNA with Mg^2+^ can achieve a positive effect on the treatment of OA by the integrated regulation of HIFs.

**Fig. 1.**
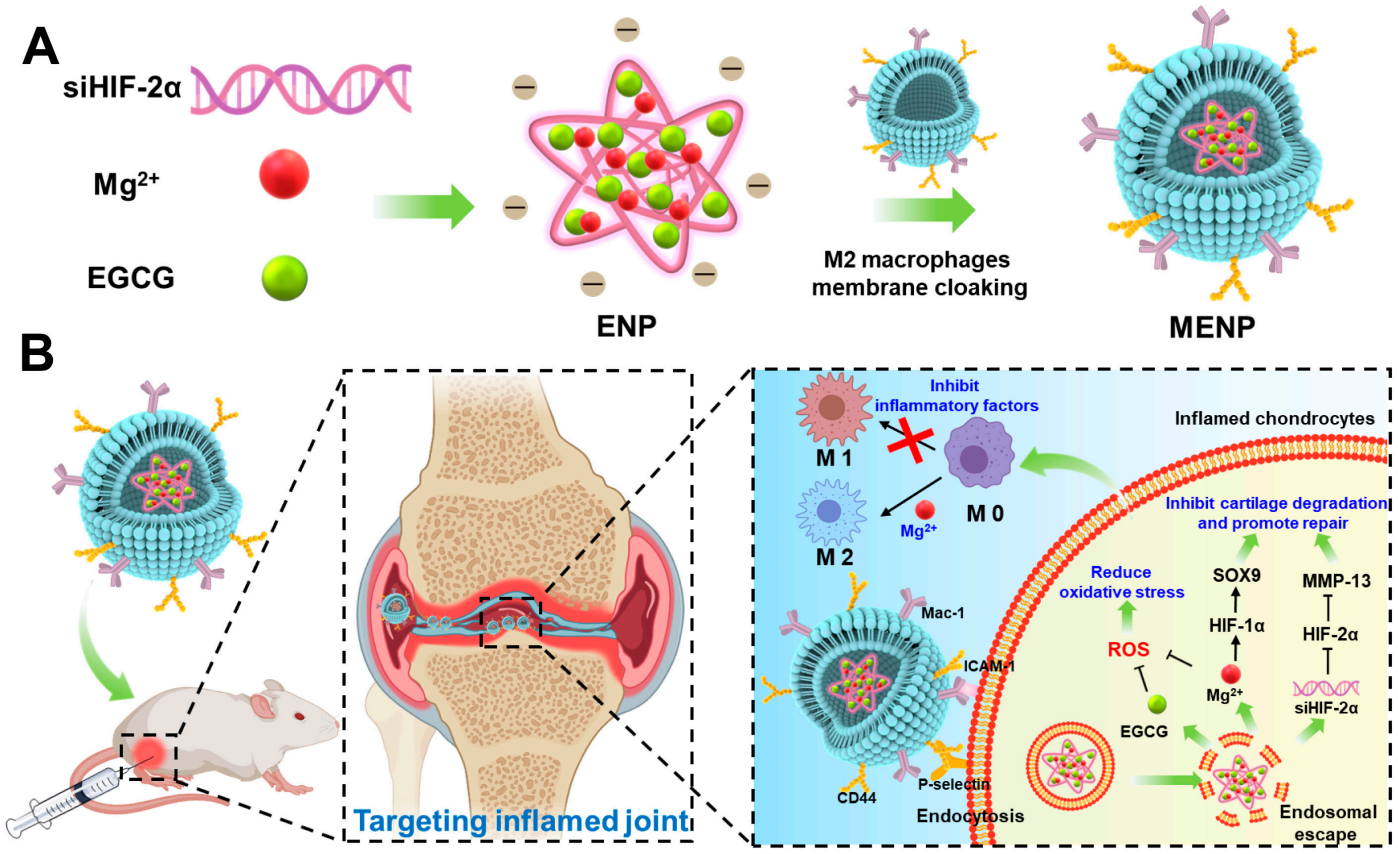
Schematic illustration of the development and use of the biomimetic gene vector MENP for treating OA. (A) Preparation of the MENP. (B) Therapeutic mechanism: The MENP effectively targets inflamed areas in OA-affected joints. It simultaneously regulates HIF-1α and HIF-2α and optimizes macrophage polarization, contributing to the effective treatment of OA.

Based on the above understandings, we herein report the biomimetic gene vector that is capable of targeted co-delivery of siHIF-2α and Mg^2+^ into inflamed cells in OA. Epigallocatechin gallate (EGCG) has recently garnered attention in drug delivery due to its excellent bioactivity and unique chemical structure [33]. EGCG has strong binding affinity with DNA, RNA, and proteins through hydrogen bond interactions, allowing it to condense siRNA into nanoparticles [34–36]. Simultaneously, EGCG and Mg^2+^ can form a complex by coordination [37], which renders EGCG the best candidate for co-loading siRNA and Mg^2+^. As depicted in Fig. 1, EGCG is assembled with HIF-2α siRNA and Mg^2+^ as the negatively charged kernel of the gene vector (ENP), followed the M2 macrophage membrane (MM) being coated on the ENP to form the biomimetic siRNA delivery system (MENP), with the goal of targeting the inflamed synovium. We then investigated the cellular absorption efficiency and gene silencing efficiency of MENP. Furthermore, we demonstrated the regulatory effects of MENP on HIF-2α and HIF-2α, as well as its effects on macrophage repolarization and anti-inflammation. Finally, we evaluated the therapeutic efficacy and safety of MENP in an OA mouse model.

## Materials and Methods

### Materials

(−)-Epigallocatechin-3-O-gallate (EGCG), magnesium chloride (MgCl_2_), ethidium bromide (EB), and water diethyl pyrocarbonate (DEPC) were purchased from Macklin Biochemical Co., Ltd. (Shanghai, China). RNase enzyme, dichloro-dihydro-fluorescein diacetate (DCFH-DA), Hoechst 33258, interleukin (IL)-1β, membrane protein extraction kit, CCK-8 kit, heparin, and Lyso-Tracker Red were purchased from Beyotime Biotechnology (Shanghai, China). Fluorescently labeled siRNA (FAM-siRNA and Cy5-siRNA), scrambled siRNA (siNC), and siHIF-2α (sense: 5′-CUCAGUUACAGCCACAUCGUCACUG-3′, antisense: 5′-CAGUGACGAUGUGGCUGUAACUGAG-3′) were purchased from GenePharma Co., Ltd. (Shanghai, China). Dulbecco modified Eagle medium (DMEM) and phosphate buffered saline (PBS) were purchased from Life Technologies (USA). Mouse F4/80, inducible nitric oxide synthase (iNOS), HIF-1α, HIF-2α, MMP-13, SOX9, tumor necrosis factor-alpha (TNF-α), IL-6, and IL-1β enzyme-linked immunosorbent assay (ELISA) kit were purchased from Elabscience Biotechnology Co., Ltd. Antibodies including PE-F4/80, PE-CCR7, FITC CD86, FITC-CD206, and APC-CD206 for cell marker labeling in a flow cytometer were purchased from BioLegend, Inc. (USA) or Cell Signaling Technology (USA). All the chemicals were used as received without further purification.

### Preparation and characterization of ENP and MENP

ENP was synthesized by gently mixing EGCG, Mg^2+^, and siRNA. Briefly, 100 μl of DEPC water of EGCG (20 μg/ml) and Mg^2+^ (20 μg/ml) were slowly mixed with 100 μl of solution of siRNA (2 μg/ml), and the reacted solution was kept at room temperature for 30 min. The interaction of EGCG with Mg^2+^ and siRNA was characterized using an EB competitive assay. When the density of RAW 264.7 cells (M0) reached 70%, 20 ng/ml IL-4 and IL-13 were added and the culture was continued for 24 h to collect M2 macrophages. The MM was extracted according to the manufacturer’s instructions of the membrane protein extraction kit. The membrane content was quantified using the BSA kit and the specific membrane proteins were verified by Western blot. The M2 membrane was stored at −80 °C for subsequent use. Subsequently, the MM from the previous step and ENP were combined (0.02 mg of M2 membrane per 2 μg of siRNA), and the 200-nm polycarbonate membrane was repeatedly passed through a micro extruder (20 times) to obtain MENP. Finally, the samples were newly prepared for use.

The size, zeta potential, and stability of ENP and MENP were determined by dynamic light scattering (Zetasizer Nano ZS90, Malvern). The morphology of ENP and MENP was further observed by transmission electron microscopy (TEM). The amount of Mg^2+^ in MENP was detected by inductively coupled plasma mass spectrometry (ICP-MS). The RNase stability of ENP and MENP was determined by agarose gel electrophoresis after incubation with RNase at 37 °C for 2 h. Furthermore, the pH-triggered siRNA and Mg^2+^ release from MENP was evaluated by incubating them in medium with or without pH 5.5 for 24 h, respectively. The FAM-siRNA fluorescence was determined by the fluorescence spectrophotometer and Mg^2+^ was detected by ICP-MS.

### Cell culture

The RAW 264.7 cells were obtained from the Type Culture Collection of the Chinese Academy of Sciences and incubated in cell culture dishes with a diameter of 10 cm containing DMEM supplemented with 10% (v/v) heat-inactivated fetal bovine serum (FBS) and 1% penicillin/streptomycin at 37 °C in a humidified atmosphere containing 5% CO_2_. Moreover, chondrocytes and synoviocytes were isolated from neonatal joint tissue and cultured in primary cultures as previously reported [38,39], with subsequent experiments performed after primary culture to the third generation. These cells were cultured in DMEM containing 4,500 mg/L glucose, 10% FBS, and 1% penicillin/streptomycin in a 5% CO_2_ at 37 °C.

### Cellular uptake and siRNA lysosomal escape

Chondrocytes and synoviocytes were seeded into 24-well plates at 5×10^4^ cells/well for 24 h, respectively. Inflamed chondrocytes and inflamed synoviocytes were induced by incubating with IL-1β (10 ng/ml) for 6 h. FAM-siRNA was used for the preparation of biomimetic siRNA delivery system. ENP and MENP were incubated with chondrocytes with or without inflammation for 6 h (100 nmol siRNA per well), respectively. The treated cells were then collected into cold PBS and monitored for FITC fluorescence using a flow cytometer. Meanwhile, the MENP-treated cells were imaged with an inverted fluorescence microscope.

The lysosomal escape of FAM-siRNA in inflamed chondrocytes was detected using a confocal laser scanning microscope (CLSM). Chondrocytes were seeded in Petri dishes (with a density of 1×10^5^ cells per dish) for 24 h and then incubated with IL-1β (10 ng/ml) for 6 h. MENP with or without Mg^2+^ was incubated with inflamed chondrocytes for 6 h. Subsequently, the cells were washed with PBS, fixed with 4% paraformaldehyde, and stained with Hoechst 33258 (10 min) and Lyso-Tracker Red (30 min). After washing again with PBS, the cells were observed under a CLSM and imaged.

### Gene silencing efficiency and therapeutic efficiency

To investigate the gene silencing efficiency of MENP, we used Western blot to evaluate the down-regulation of HIF-2α protein. Briefly, chondrocytes were seeded into 6-well cell culture plates at a density of 1×10^6^ cells/well and treated with IL-1β for 12 h. Then, MENP and ENP containing 1 μmol of siHIF-2α, as well as Mg^2+^ and MM corresponding to MENP, were added, respectively, and PBS was used as a negative control. Afterwards, cells were harvested and lysed at the end of the 24-h transfection. The protein was separated by sodium dodecyl sulfate–polyacrylamide gel electrophoresis and transferred to polyvinylidene fluoride (PVDF) membranes by electrophoresis. The PVDF membranes were blocked in 5% skim milk and then incubated with primary antibody overnight at 4°C. Subsequently, the corresponding anti-rabbit or anti-mouse secondary antibody with horseradish peroxidase (HRP) was incubated with the PVDF membrane. Finally, ECL-prime was used to detect the membrane.

The CCK-8 kit was used to investigate the effect of MENP on the proliferation of inflammatory chondrocytes. Specifically, chondrocytes were seeded into a 96-well cell culture plate at 5,000 cells/well for 24 h and then treated with IL-1β for 12 h. Thereafter, MENP and ENP containing 50 nmol of siHIF-2α, as well as Mg^2+^ and MM complete medium corresponding to MENP, were added, and PBS was used as a negative control. At the end of 48 h of incubation, CCK8 solution was added to each well. Absorption intensity changes of formazan were measured using a microplate reader at 460 nm, and the cell survival rate was evaluated. Subsequently, the intracellular reactive oxygen species (ROS) level was analyzed by DCFH-DA and flow cytometry analysis, and the cell viability was measured by a CCK-8 kit according to the manufacturer’s instructions.

To investigate the effect of Mg^2+^ on HIF-1α and SOX9 expression in inflammatory chondrocytes, Western blot was used according to protocols as previously described above. Chondrocytes were grown on coverslips, proinflammatory with IL-1β and treated with Mg^2+^ or MENP, respectively. Subsequently, cells were fixed using freshly prepared 4% paraformaldehyde, permeabilization with Triton X-100, and blocking in BSA. The cells were incubated with the anti-SOX9 primary antibody and then incubated with secondary antibody (Alexa Fluor 555). The coverslips were then mounted with 4′,6-diamidino-2-phenylindole (DAPI) Fluor-Gel on glass slides. Cells were observed and scanned using a confocal microscope.

### Macrophage polarization and oxidative stress in vitro

The effect of in vitro inflammatory cells on macrophage polarization was analyzed by immunostaining [40]. Briefly, synoviocytes were seeded into the upper compartment of the chambers at a density of 5×10^4^ cells/dish for 12 h and then treated with IL-1β for 12 h. Thereafter, different formulations including PBS, Mg^2+^, and MENP (100 nmol siRNA and 7 μg Mg^2+^ per well) were added to upper chambers and co-cultured with macrophages in the lower chamber. After incubation for 24 h, the synoviocytes in the upper face were removed, and the macrophages in the lower chamber were stained using the PE-CCR7 antibody and the FITC-CD206 antibody. Then, cells were washed with cold PBS containing heparin for 3 times and observed by CLSM. In addition, the intracellular ROS level of the macrophage in the lower chamber was analyzed by DCFH-DA and flow cytometry analysis, and the levels of IL-6, TNF-α, and Mg^2+^ in the culture medium were also determined by ELISA and ICP-MS.

In addition, the mRNA levels of M1 macrophage-specific gene (Nos2) and M2 macrophage-specific gene (Arg1) in RAW 264.7 cells were monitored by real-time PCR. RAW 264.7 cells received the same treatment and IL-1β challenge as described above, and the mRNA levels were determined by real-time PCR. Results were presented as percentage mRNA levels of the normal control (NC) or the MENP group. Sequences of primers are as follows: Nos2 (forward: 5′-TGGGACAGCACAGAATGTT-3′; reverse: 5′-GAAATCCGATGTGGCCTTGTG-3′) and Arg1 (forward: 5′-CAAAAGGACAGCCTCGAGGA-3′; reverse: 5′-GGAGCTGTCATTAGGGACATC-3′). Furthermore, the ROS level of macrophage was analyzed by DCFH-DA. Treated macrophages as described above were subjected to flow cytometry analysis.

### Establishment of the OA model and treatment grouping

Male C57/BL6 mice aged 3 months (*n* = 20) were purchased from the Guangdong Medical Experimental Animal Center. The animal experiment was approved by the Animal Ethics and Experimentation Committee of Jinan University (IACUC-20220617-02). OA modeling was induced by surgically severing the anterior cruciate ligament with partial medial meniscectomy [41]. Subsequently, the experimental animals were randomly assigned into 4 groups (*n* = 5 each): (a) NC, (b) OA group, (c) ENP group, and (d) MENP group. The formulations administered included 300 μg/kg siHIF-2α, 750 μg/kg Mg^2+^, and 3 mg/kg EGCG. At relative early stage of OA (week 2 after operation), different formulations were injected into the OA knee joint according to groups, once a week for 4 consecutive weeks. The mice were anesthetized and euthanized by cervical dislocation on day 42 after surgery.

### Intra-articular retention effect

Cy5-siRNA was used for the preparation of ENP and MENP to determine their retention time in OA knee joint. At 1, 6, 12, 24, and 48 h after injection, mice were anesthetized and imaged using a Bruker NirVivo animal imaging system for each time point at excitation/emission, 745 nm/805 nm.

### In vivo anti-inflammation efficiency

At week 6 after treatment, mice were euthanized and joint tissues were harvested. After tissue lysis for supernatants, HIF-2α, MMP-13, TNF-α, IL-6, and IL-1β levels in cartilage and synovium were determined by ELISA. The joint tissues were fixed in 4% paraformaldehyde and decalcified in 9% formic acid. Paraffin sections were analyzed by staining with Safranin O/fast green or hematoxylin and eosin (H&E). HIF-2α and MMP-13 protein were visualized by immunofluorescence (IF) staining, HIF-1α and SOX9 protein were analyzed by immunohistochemical (IHC) staining. Primary antibodies were applied and incubated overnight at 4 °C. HRP-conjugated secondary antibodies were applied and incubated for 1 h at room temperature. For IHC staining, the signal was developed with the DAB kit (TA-060-QHDX, Thermo Fisher Scientific) and counterstained with hematoxylin. For IF staining, DAPI (D1306, Life Technology) was used to stain the nuclei.

### Macrophage polarization in vivo

For the investigation of macrophage polarization in vivo, IF and flow cytometry were carried out. F4/80 and iNOS were analyzed by IF staining as in the previous method. Moreover, cartilage and synovium tissues were digested with collagenase D and DNase 1 for 60 min at 37 °C and filtered through a silk mesh to obtain cell suspensions. The cell suspensions were labeled with PE-F4/80, APC-CD206, and FITC-CD86 for flow cytometry according to the manufacturer’s instructions.

### Statistical analysis

All data were presented as mean ± standard deviation, and statistical analysis was performed using the Student’s *t* test. Differences between 2 groups were judged to be significant at *P < 0.05 and very significant at **P < 0.01 and ***P < 0.001.

## Results

### Preparation and characterization of MENP

**Fig. 2.**
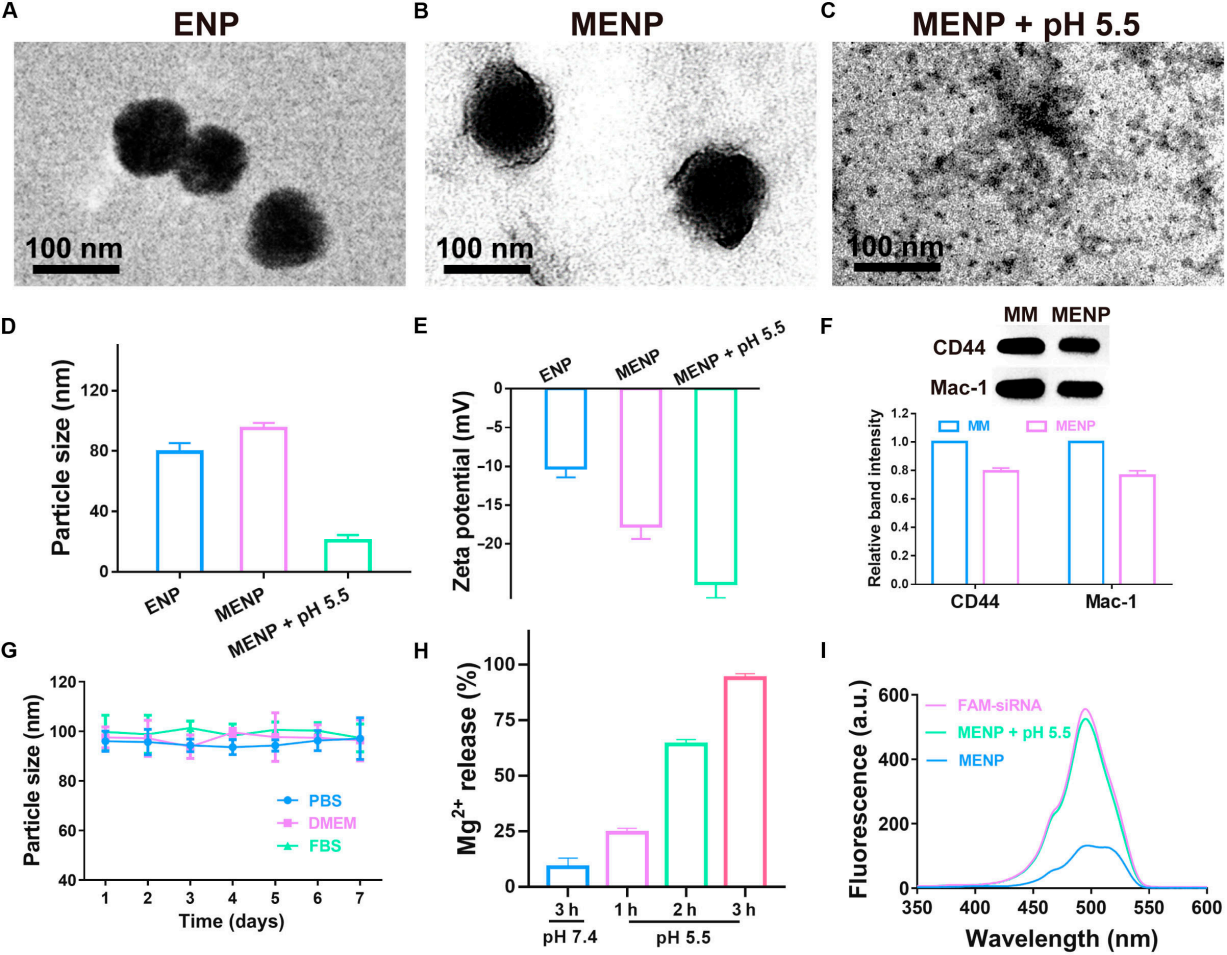
Preparation and characterization of MENP. (A) TEM image of ENP, (B) MENP, and (C) MENP at pH 5.5. Scale bar: 100 nm. (D) Hydrodynamic sizes of ENP, MENP, and MENP at pH 5.5 by DLS measurement. (E) Zeta potentials of ENP, MENP, and MENP at pH 5.5. (F) Western blot of CD44 and Mac-1 in MM or MENP. (G) Change in size of MENP in different media over 1 week. (H) MENP release behavior of Mg^2+^. (I) Fluorescence spectra of FAM-siRNA, MENP, and MENP at pH 5.5 (excitation/emission, 490/520 nm).

Based on the goal of simultaneous regulation of HIF-1α and HIF-2α, we constructed the biomimetic gene vector in 3 steps using EGCG, Mg^2+^, siRNA, and MM. First, ENP was prepared via cooperative hydrogen bond, hydrophobic interactions, and coordination. The mass ratio of EGCG, Mg^2+^, and siRNA in ENP was optimized at 10:10:1. Subsequently, the MM is extracted. Ultimately, ENP was covered with macrophage membranes to obtain MENP. The interactions of EGCG with Mg^2+^ and siRNA were confirmed by EB competitive binding and TEM experiments. EGCG with Mg^2+^ significantly decreases the intensity of fluorescence yielded by the complexation of EB with siRNA (Fig. S1), suggesting that EGCG with Mg^2+^ can successfully complex siRNA. Moreover, as shown in Fig. 2A and B, the ENP exhibited a relatively uniform round shape and MENP morphology was spherical core–shell according to TEM. Similarly, the size of MENP was larger compared to ENP (95 nm vs. 79 nm), corresponding to the addition of a cell membrane onto ENP, which was the same as previously published reports on macrophage membrane thickness [42]. The surface zeta potential decreased from −10.2 to −17.7 mV after MM coating (Fig. 2D and E). These results confirm that MENP is successfully prepared.

The MENP maintained a similar presence of surface proteins, such as adhesion factors that target inflammation (e.g., CD44 and Mac-1). To identify 2 potential proteins, CD44 and Mac-1, MENP was subjected to Western blot. As illustrated in Fig. 2F, the expression of CD44 and Mac-1 was maintained in the MENP, similar to that of MM. The stability of MENP was detected as shown in Fig. 2G and is excellent in a variety of media over a period of 7 days. Interestingly, MENP became fragmented at pH 5.5, which would facilitate the lysosomal escape of siRNA (Fig. 2C). Further, we investigated the pH-dependent release of Mg and siRNA. Around 8.8% of Mg^2+^ was released from MENP within 3 h after exposure to PBS. Notably, MENP exhibited a significantly enhanced release of Mg^2+^ at pH 5.5, achieving nearly 93.8% release efficiency of Mg^2+^ at the same time interval (Fig. 2H). Similarly, the release of siRNA is pH responsive, as shown in Fig. 2I.

### Ability of MENP to deliver siRNAs extra-/intracellularly

**Fig. 3.**
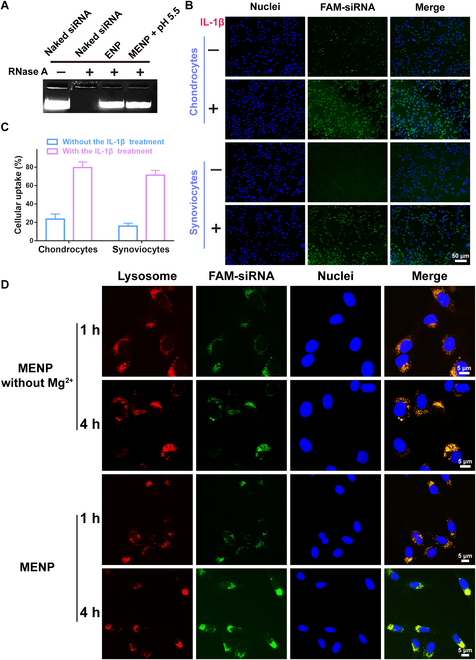
Ability of MENP to deliver siRNAs extra-/intracellularly. (A) Stability of MENP complexes against RNase. (B) Fluorescence images of chondrocytes and inflamed synoviocytes (normal or inflamed) incubated with MENP. Green fluorescence: FAM-labeled siRNA. Scale bar: 50 μm. (C) Quantitative analysis of cellular uptake of MENP by a flow cytometer. (D) Intracellular distribution of FAM-siRNA. Red fluorescence: lysosomes stained with Lyso-Tracker Red. Green fluorescence: FAM-siRNA. Blue fluorescence: nuclei stained with Hoechst 33258. Scale bar: 5 μm.

The MENP complex efficiently prevents the degradation of siRNA by RNase when MENP is delivered in vivo and liberates siRNA at pH 5.5 (Fig. 3A). To investigate the internalization of MENP in chondrocytes and synoviocytes with or without inflammation, FAM was used to label MENP. Compared with normal cells, inflammatory chondrocytes and synoviocytes showed stronger fluorescence intensity (Fig. 3B), suggesting that they take up MENP more efficiently. We further quantified the internalization of ENP and MENP through flow cytometric analysis. As illustrated in Fig. 3C, the uptake level of MENP into activated chondrocytes was fourfold higher than that into normal chondrocytes (Fig. 3C), while ENP showed similar uptake levels into activated chondrocytes and normal chondrocytes (Fig. S2A), with the same result for synoviocytes. Notably, the MENP displayed a higher internalization within activated chondrocytes and synoviocytes than ENP, further revealing that the targeting effect of MENP to inflamed lesions may be attributed to the specific ligands on the MM. Additionally, as shown in Fig. S2B, MENP demonstrated significantly lower uptake by Raw264.7 macrophages (approximately 13%) compared to ENP (approximately 66%), highlighting the efficacy of macrophage membrane cloaking in reducing macrophage phagocytosis.

The ability of MENP to efficiently deliver siRNA into the cytosol for gene silencing is crucial. Therefore, we monitored the intracellular trafficking of MENP containing FAM-labeled siRNA, with a particular focus on endosomal localization, using a CLSM. Moreover, to assess the colocalization of FAM-siRNA and Lyso-Tracker, the Manders coefficients were calculated using ImageJ software, with larger values indicating more siRNA being sequestered in the endosome/lysosome [43]. From the CLSM merged images presented in Fig. 3D, MENP without Mg^2+^ exhibited no green pixels at 1 h and 4 h, while the Manders coefficients remained high (Fig. S3). This observation suggests that the major fraction of MENP without Mg^2+^ has been completely trapped in the lysosome. In contrast, the colocalization ratio of MENP was notably lower than those of the MENP without Mg^2+^, indicating that the major faction of MENP might escape from the endosome, a finding consistent with our CLSM merged images. This ability of MENP to escape the endosome/lysosome may be attributed to the release of Mg^2+^ in the acidic lysosome environment, disrupting the osmotic pressure balance and leading to lysosomal rupture.

### In vitro anti-inflammation efficiency of MENP

**Fig. 4.**
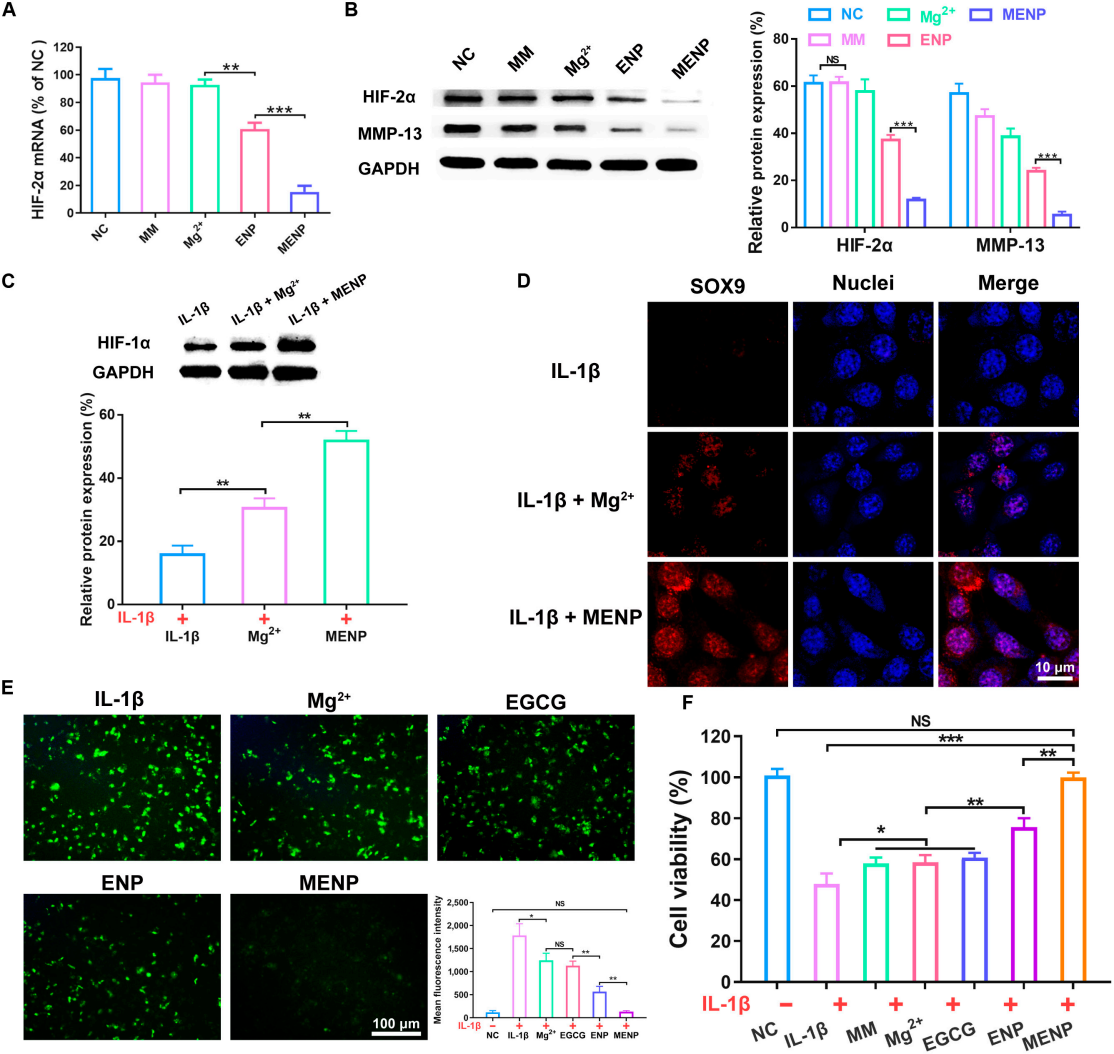
In vitro anti-inflammation efficiency of MENP. (A) Relative HIF-2α mRNA levels in inflammatory chondrocytes. (B and C) Expression levels of HIF-2α, MMP-13, and HIF-1α as determined by Western blot. (D) The nuclear expression of SOX9 in inflammatory chondrocytes detected with fluorescent immunocytochemistry. Scale bar: 10 μm. (E) The intracellular ROS levels detected with DCFH-DA probe. Scale bar: 100 μm. (F) The cellular viability of inflammatory chondrocytes incubating MENP. Data are shown as mean ± standard deviation (*n* = 3). ***P < 0.001, **P < 0.01, *P < 0.05.

The experimental results described above demonstrated that MENP exhibits exceptional endocytosis properties and a remarkable ability to escape lysosomes, thereby facilitating gene transfection. We first assessed the efficiency of siHIF-2α-mediated gene silencing by measuring HIF-2α mRNA levels in IL-1β-challenged chondrocytes. As illustrated in Fig. 4A, MENP demonstrated a substantial 85% reduction in HIF-2α mRNA levels, surpassing ENP (40%), largely attributed to the presence of the macrophage membrane coating. In contrast, MM and Mg^2+^ had negligible effects on the HIF-2α mRNA level. Consistent outcomes were observed at the HIF-2α protein level, as confirmed by Western blot analysis (Fig. 4B). In the presence of pro-inflammatory cytokines, HIF-2α promotes MMP-13 expression, leading to deterioration of the extracellular matrix and cartilage damage. As shown in Fig. 4B, MENP markedly suppressed MMP-13 expression compared to other treatment groups, suggesting a protective effect against cartilage degradation and highlighting the potential therapeutic benefits of MENP in mitigating OA-associated pathology.

Subsequently, we further investigated the effect of Mg^2+^ on the expression of HIF-1α in chondrocytes; HIF-1α could potentially contribute to the enhanced matrix synthesis of cartilage and the alleviation of OA progression. Figure 4C shows that the expression of HIF-1α in cartilage tissue of the Mg^2+^ group was higher than that of the control group, indicating a therapeutic potential for Mg^2+^. Furthermore, MENP significantly enhanced the regulation of HIF-1α by Mg^2+^. Simultaneously, MENP markedly elevated the expressions of SOX9 in inflammatory chondrocytes (Fig. 4D), which is required to maintain the chondrocyte differentiation process. In summary, MENP exerts protective effects on cartilage by down-regulating HIF-2α and MMP-13 via siHIF-2α, while simultaneously enhancing cartilage matrix protection through the up-regulation of HIF-1α and SOX9 via Mg^2+^. These combined actions hold promise for effectively preserving cartilage integrity and combating destructive processes in OA.

To investigate oxidative stress, we conducted an assessment of ROS levels in inflamed chondrocytes using the DCFH-DA probe after treatment with various formulations. Figure 4E and Fig. S4 revealed that the intracellular ROS levels were elevated when cells were incubated in IL-1β-containing media. Notably, ENP exhibited resistance to ROS due to the anti-inflammatory effects of EGCG. Importantly, MENP markedly decreased the expression of ROS compared with ENP, which is consistent with the results of flow cytometry analysis. These results demonstrated that the excellent targeted uptake ability and anti-oxidative stress capacity allow MENP to enhance the regulation of HIF-1α by Mg^2+^. Furthermore, cell activity assay revealed that MENP could effectively prevent chondrocyte death induced by IL-1β (Fig. 4F). Moreover, different formulations showed negligible cytotoxicity to chondrocytes, indicating their desired cytocompatibility (Fig. S5). These observations collectively support the potential therapeutic utility of MENP in mitigating oxidative stress-related mechanisms and preserving chondrocyte viability in the context of OA pathology.

### Macrophage repolarization by MENP

**Fig. 5.**
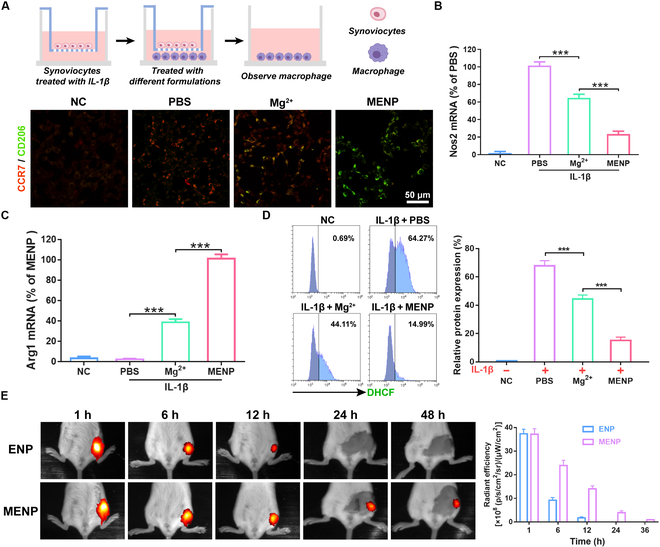
MENP-mediated macrophage polarization in vitro and the retention time of MENP in joint cavity. (A) Illustration of the transwell experiment for evaluating the indirect regulatory effects of MENP on macrophage. Activated synoviocytes and MENP were added to the upper chamber to be co-cultured with macrophages in the lower chamber for 24 h. Fluorescence images of RAW 264.7 cells immunostained for CCR7 and CD206. Scale bar: 50 μm. (B and C) Relative Arg1 and Nos2 mRNA levels in RAW 264.7 cells. (D) The intracellular ROS levels of RAW 264.7 cells. (E) Fluorescence images of OA mice at different time points after intra-articular injection. Data are shown as mean ± standard deviation (*n* = 3). ***P < 0.001.

Our goal was to determine whether activated synoviocytes could induce the polarization of macrophages from an M0 to an M1 phenotype through a transwell experiment (Fig. 5A). We also investigated whether M1 macrophages could be converted into anti-inflammatory M2 macrophages after treatment with MENP. By staining with anti-CD206 (M2 biomarker, green fluorescence) and anti-CCR7 (M1 biomarker, red fluorescence) and evaluating macrophage polarization through CLSM images, we gained insights into these processes. Figure 5A provides clear evidence of M1 polarization occurring after co-culture with inflamed synoviocytes. However, RAW 264.7 cells (M0 macrophages) were repolarized to M2 macrophages when exposed to inflamed synoviocytes after treatment with MENP. Notably, MENP had a stronger and more effective impact compared to Mg^2+^. Consistent with the above findings, after treatment with MENP, the Nos2 (M1 macrophage-specific gene) mRNA level was decreased by around 77% (Fig. 5B), while the Arg1 (M2 macrophage-specific gene) mRNA level was increased by around 43-fold (Fig. 5C). In addition, as shown in Fig. 5D, inflamed synoviocytes induced macrophage activation to produce intracellular ROS, while treatment with MENP normalized ROS level. The obtained results substantiated the capability of MENP in polarizing macrophages from the proinflammatory M1 phenotype to the anti-inflammatory M2 phenotype through its influence on synoviocytes.

To investigate the indirect effect of MENP on macrophage polarization, we evaluated the levels of inflammatory factors and Mg^2+^ in the culture medium. Mg^2+^ is recognized for its ability to facilitate the conversion of macrophages from an M1 to an M2 phenotype. As illustrated in Fig. S6, Mg^2+^ reduced the release of inflammatory factors from inflamed synoviocytes. Importantly, MENP exhibited a more pronounced reduction in inflammatory factor levels compared to magnesium ions alone. Furthermore, analysis of the MENP-treated medium revealed the presence of Mg^2+^, indicating that MENP exerts its effect by inhibiting M1 macrophage polarization through suppression of inflammatory factors while concurrently promoting M2 macrophage polarization mediated by magnesium ions.

### Evaluation of joint cavity residence

To assess the in vivo retention time of MENP, we intra-articularly injected MENP containing Cy5-siRNA into the OA knee joints. Over a 48-h span, we monitored fluorescence within the joint using an in vivo imaging system (Fig. 5E). Both ENP and MENP exhibited a decline in intensity and area over time, with the ENP group showing nearly undetectable fluorescence at the 12-h mark, suggesting a limited retention effect. In contrast, the fluorescence intensity of MENP decreased at a slower rate and was still observable 48 h after intra-articular injection. The result demonstrated that MENP has long-term retention in the OA joint, primarily due to the protective cloak of macrophage membranes. Such a feature has the potential to obviate the need for repeated injections, thereby optimizing therapeutic efficacy.

### In vivo therapeutic efficacy of MENP

**Fig. 6.**
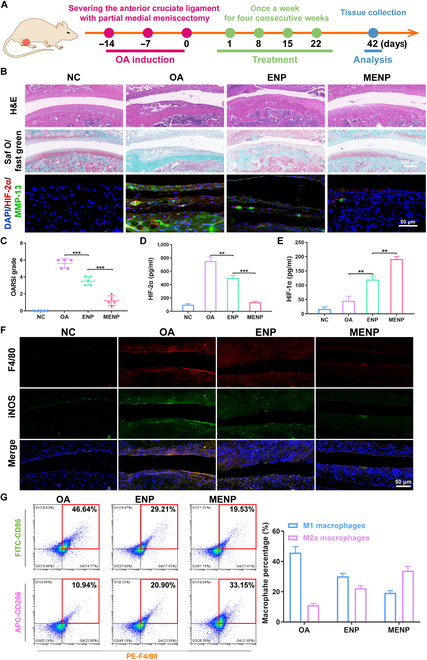
In vivo therapeutic efficacy of MENP in OA. (A) Illustration of the study protocol for OA modeling and the therapeutic regimen. (B) Representative images of H&E, safranin-O/fast green staining, and IF staining of HIF-2α (red fluorescence) and MMP-13 (green fluorescence) in cartilage sections after different treatments. Scale bar: 100 μm. (C) Quantitative analysis of cartilage degeneration and damage by the OARSI scoring system. (D and E) The expression of HIF-1α and SOX9 in cartilage and synovium. (F) Representative images of immunofluorescence of F4/80 and iNOS in the OA joint after different treatments. Scale bar: 50 μm. (G) Quantification of M1/M2 macrophages from the OA joint by flow cytometry. Data are shown as mean ± standard deviation (*n* = 3). ***P < 0.001, **P < 0.01.

To investigate the therapeutic efficiency of MENP in vivo, the OA model was established and the treatment was administered for 4 consecutive weeks at weekly intervals (Fig. 6A). The assessment of therapeutic efficacy and underlying mechanisms of MENP involved a range of analyses, including histology, immunohistochemistry, and IF. In the OA joint, cartilage degradation was strikingly evident, as highlighted by safranin O/fast green staining, demonstrating severe wear and deterioration of the cartilage surface (Fig. 6B). In the ENP-treated knees, there was a notable reduction in cartilage erosion, resulting in a smoother cartilage surface with fewer clefts. Encouragingly, knees treated with MENP exhibited only a slight decrease compared to the NC. The Osteoarthritis Research Society International (OARSI) score in the OA group increased due to significant cartilage surface abrasion and focal loss of full-thickness cartilage. Moreover, ENP reduced cartilage destruction and OARSI scores compared to OA. However, the mice from the MENP group showed the lowest OA score (Fig. 6C), signifying the significant therapeutic potential of MENP in OA. Furthermore, we investigated the influence of MENP on the regulation of HIFs in the OA joint. As shown in Fig. 6B and D, MENP remarkably diminished the expression of HIF-2α as compared with the other groups, displaying a similar inhibitory effect on MMP-13 expression (Fig. S7). Contrastingly, the expression of HIF-1α and SOX9 was enhanced due to the regulatory effect of Mg^2+^ in the MENP group (Fig. 6E and Fig. S8). Additionally, we measured the levels of TNF-α, IL-1β, and IL-6 in the OA joint by ELISA measurements, which are known to be elevated with the onset of arthritis and correlate with its severity. We found that the expression of IL-6, TNF-α, and IL-1β was slightly decreased in ENP groups, while they were significantly reduced in the MENP group with respect to the OA group (Fig. S9). These results indicated that MENP enabled OA to progress toward rehabilitation by modulating HIFs (down-regulating HIF-2α and up-regulating HIF-1α) and effectively inhibiting the release of immune inflammatory factors.

To investigate whether MENP could suppress M1 macrophage polarization in vivo, we examined the accumulation of M1 macrophage within the context of OA following treatment. The results of immunofluorescent staining revealed a significant increase in iNOS (M1-like macrophage marker) within F4/80 (macrophage marker)-positive cells in the OA group. Conversely, the percentage of iNOS-expressing cells within F4/80-positive cells was substantially reduced in the MENP-treated groups compared to the ENP group (Fig. 6F). To further dissect the phenotypic characterization of macrophages in OA, M1 macrophages (FITC-CD86) and M2 macrophages (APC-CD206) from synovial tissue were fluorescence-labeled and analyzed via flow cytometry (Fig. 6G). As anticipated, Fig. 6G exhibited the highest proportion of M2 macrophages (CD206 positive) and the lowest proportion of M1 macrophages (CD86 positive) in comparison to other treatment groups. These findings highlight the independent effect of MENP in promoting M2 polarization while inhibiting M1 polarization of macrophages, suggesting a potential therapeutic benefit for OA.

## Discussion

The development of effective therapeutic strategies for OA remains a critical challenge in clinical settings. In this study, we engineered a novel biomimetic gene vector termed MENP with the goal of concurrently regulating key factors involved in OA pathogenesis, namely, HIF-1α, HIF-2α, and macrophage polarization. Through a comprehensive in vitro and in vivo evaluation, we demonstrated that MENP exhibits remarkable potential for mitigating OA progression by targeting multiple pathological pathways simultaneously.

MENP was strategically designed to capitalize on the unique properties of its constituent components. The core of MENP comprised EGCG, Mg^2+^, and siRNA, which were encapsulated within ENP utilizing cooperative hydrogen bonding, hydrophobic interactions, and coordination chemistry. This design facilitated the stable and efficient encapsulation of siRNA, essential for targeted gene silencing. Subsequent encapsulation with MM endowed MENP with a multifunctional outer shell that not only provided camouflage to evade macrophage phagocytosis but also imparted specific ligands (CD44 and Mac-1) crucial for targeting inflamed tissues, such as those found in OA joints [23,24].

The efficacy of MENP in delivering siRNA into chondrocytes and synoviocytes was evidenced by its superior cellular uptake in inflamed cells compared to normal cells. This selective internalization was attributed to the specific targeting ligands present on the MM-coated surface of MENP. Furthermore, MENP demonstrated efficient endosomal escape, which was facilitated by the pH-responsive release of Mg^2+^ within acidic lysosomal environments. This capability prevented siRNA entrapment within endosomes/lysosomes, enabling cytosolic delivery and effective gene silencing.

The therapeutic efficacy of MENP in OA stems from its multifaceted actions involving targeted gene silencing, cartilage protection, anti-inflammatory modulation, and macrophage repolarization.

MENP efficiently delivers siHIF-2α to inflamed chondrocytes, resulting in a substantial reduction (85%) in HIF-2α mRNA levels. Suppressed HIF-2α expression by MENP leads to a significant decrease in MMP-13 expression, mitigating extracellular matrix degradation and cartilage damage, key factors dampening OA progression [44]. Conversely, MENP promoted the expression of HIF-1α and SOX9 through Mg^2+^ mediation, critical for cartilage matrix maintenance and chondrocyte differentiation [44–47]. This dual action of MENP in modulating HIF-1α/2α levels underscores its potential in preserving cartilage integrity and attenuating OA-associated pathology.

Oxidative stress has been implicated in exacerbating inflammation and worsening OA progression [32]. MENP effectively reduces intracellular ROS levels in inflamed chondrocytes through EGCG, thereby mitigating oxidative stress. The reduction in inflammatory factor release by MENP signifies its potent anti-inflammatory effects, which contribute to the attenuation of OA-associated inflammation.

Macrophage infiltration and polarization within the synovium have garnered considerable attention due to the pivotal role of macrophages in the inflammatory cascade underlying OA. The distinct phenotypes of macrophages influence the local inflammatory milieu within OA joint cavities [48–50]. Consequently, modulating macrophage polarization is deemed crucial for effective arthritis treatment. To investigate the indirect regulatory effects of MENP on macrophage in OA, we examined the phenotypic characterization of macrophage after co-culture with IL-1β-activated synoviocytes. A important observation was that MENP promotes the repolarization of M1 macrophages toward an anti-inflammatory M2 phenotype through interactions with inflamed synoviocytes. The down-regulation of M1-associated genes (Nos2) and up-regulation of M2-associated genes (Arg1) highlight its capacity to modulate macrophage polarization. The observed repolarization was mediated by the interaction between MENP and inflamed synoviocytes, leading to reduced expression of inflammatory mediators and decreased macrophage intracellular ROS levels. Furthermore, MENP facilitated the release of Mg^2+^, known for their role in promoting M2 macrophage polarization [51,52], thereby augmenting its therapeutic impact on macrophage-related inflammation within OA joints.

The in vivo efficacy of MENP was evaluated using an OA mouse model, demonstrating substantial improvements in cartilage integrity and disease severity following intra-articular administration. The strategic coating of nanoparticles with macrophage membranes has been highlighted in prior studies, emphasizing its capacity to enhance adhesion and accumulation within inflamed cartilage, thereby extending the agent’s bioavailability [25]. MENP demonstrates extended intra-articular retention, facilitating sustained therapeutic effects within the OA joint. Histological analyses demonstrated that joints treated with MENP exhibited markedly reduced cartilage erosion and MMP-13 expression in comparison to control groups. Furthermore, MENP effectively modulated the expression of HIFs and inflammatory factors within the OA joint environment. Notably, MENP suppressed M1 macrophage accumulation while promoting M2 macrophage polarization within OA synovial tissue, thus fostering an anti-inflammatory microenvironment and highlighting its comprehensive therapeutic potential in the context of OA pathology. These findings underscore the promise of MENP as a versatile nanomedicine approach for targeted and multifaceted OA therapy.

### Conclusion

In the current study, the biomimetic gene vector MENP was developed to co-deliver siHIF-2α, Mg^2+^, and EGCG to the inflamed regions within OA-affected joints. MENP offers a multifaceted approach to mitigating inflammation and expediting cartilage healing by concurrently regulating HIFs (HIF-1α and HIF-2α) and modulating macrophage polarization. The investigation revealed robust cellular uptake properties of MENP in vitro and notable inflammatory targeting efficacy in vivo. Importantly, MENP demonstrated effective silencing of HIF-2α while enhancing HIF-1α-mediated cartilage restoration, aided by the inclusion of Mg^2+^. Moreover, MENP exhibited the unique ability to drive macrophage polarization toward the anti-inflammatory M2 phenotype through interactions with inflamed synoviocytes. In summary, MENP emerges as a promising therapeutic strategy for OA, offering the potential to alleviate inflammation, promote resolution, and facilitate tissue repair.

## Ethical Approval

All procedures were approved by the Animal Ethics and Experimentation Committee of Jinan University (IACUC-20220617-02).

## Data Availability

The datasets supporting the conclusions of this article are included within the article and the Supplementary Materials.
