# Supplementary material for: Simultaneously Modulating HIF-1α and HIF-2α and Optimizing Macrophage Polarization through the Biomimetic Gene Vector toward the Treatment of Osteoarthritis
Source: Biomater Res. 2024 Jul 29;28:0059. doi: 10.34133/bmr.0059 (PMC11283864; doi:10.34133/bmr.0059)
Supplement: Supplementary 1 — Figs. S1 to S9 [file bmr.0059.f1.docx]

Supporting Information

Simultaneous Modulating HIF-1α and HIF-2α and Optimizing Macrophage Polarization though the Biomimetic Gene Vector toward the Treatment of Osteoarthritis

*Boyuan Zheng^a^, Yiwan Shi^a^, Lei Xiao^a^, Bowei Li^a^, Zihang Chen^b^, Jing Zhao^d,e^, Shaoping Li^d,e^, Huige Hou^a^, Jieruo Li^a^, Huajun Wang*^,a^, Peng Wu*^,b^, Xiaofei Zheng*^,a^*

a Department of Sports Medicine, The First Affiliated Hospital, Guangdong Provincial Key Laboratory of Speed Capability, The Guangzhou Key Laboratory of Precision Orthopedics and Regenerative Medicine, Jinan University, 510630, Guangzhou, China.

b Department of Orthopedics, Shanghai Tenth People’s Hospital, Tongji University School of Medicine, 200072, Shanghai, China.

c Department of psychology, Li Ka Shing Faculty of Medicine, State Key Laboratory of Brain and Cognitive Sciences, The University of Hong Kong, Hong Kong SAR, China.

d Joint Laboratory of Chinese Herbal Glycoengineering and Testing Technology, University of Macau & National Glycoengineering Research Center, China.

e State Key Laboratory of Quality Research in Chinese Medicine, Institute of Chinese Medical Sciences, Department of Pharmaceutical sciences, Faculty of Health Sciences, University of Macau, China.

*E-mail: zhengxiaofei12@163.com, wupeng03010814@163.com, and whj323@126.com


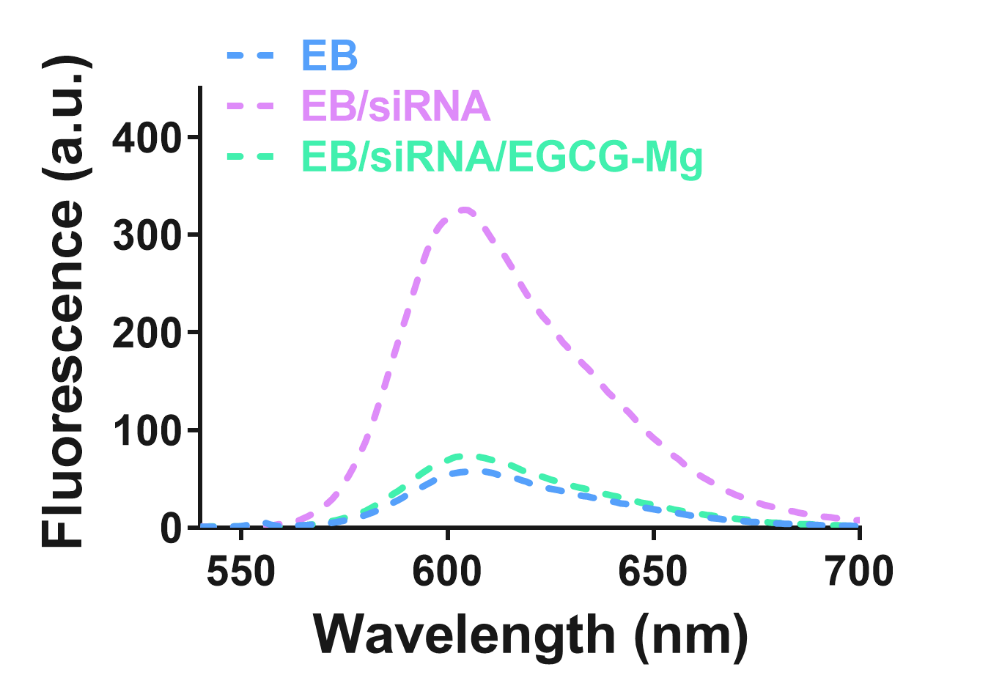


**Figure S1.** EB competitive binding assay. The binding of EGCG to siRNA-Mg causes the exclusion of EB from siRNA and the quenching of EB fluorescence.


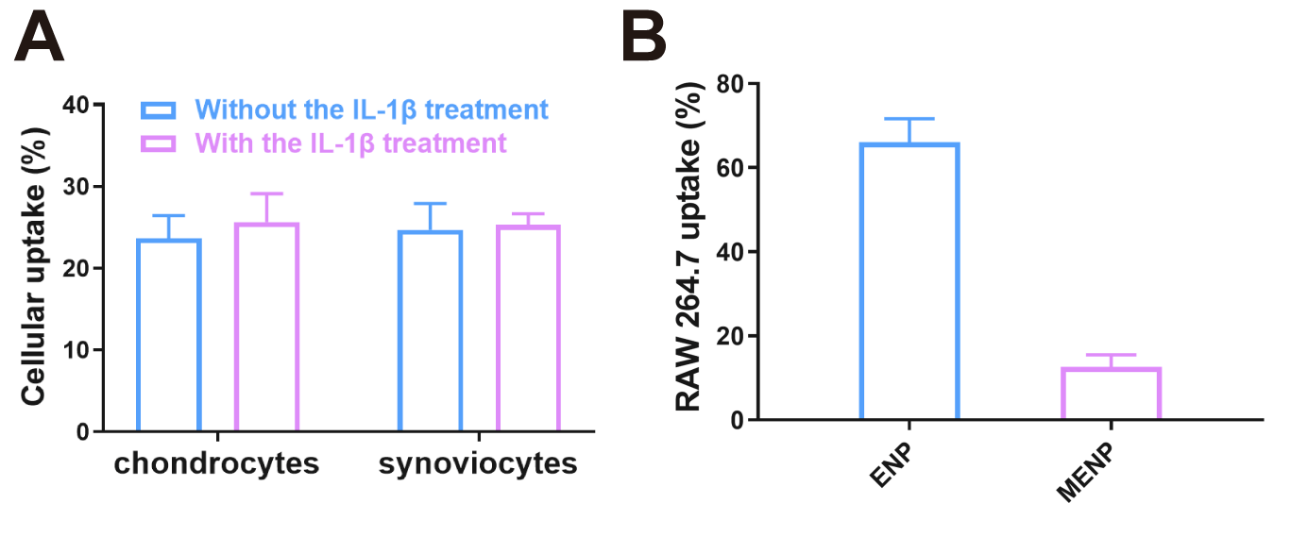


**Figure S2.** (A) Quantitative analysis of cellular uptake of ENP by Flow cytometer. (B) RAW 264.7 cell uptakes efficiency of ENP and MENP.


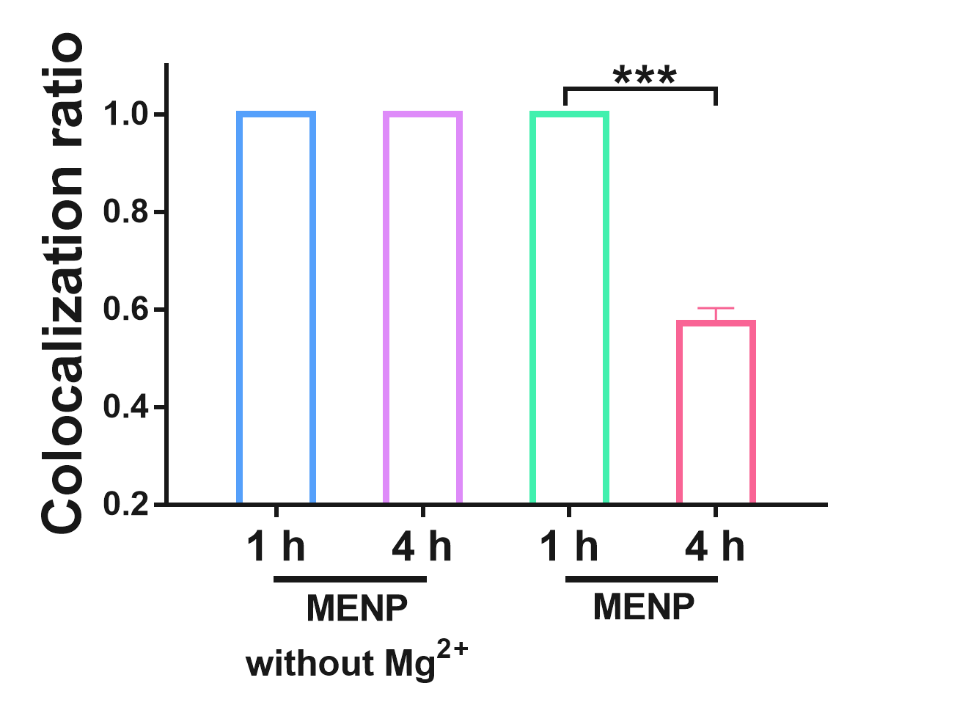


**Figure S3.** Colocalization ratios of MENP with or without Mg^2+^. ***P < 0.001.


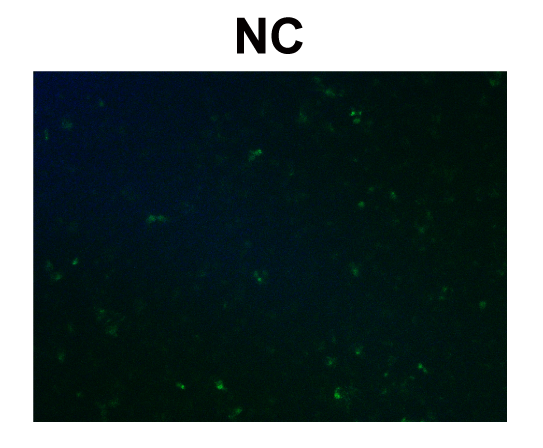


**Figure S4.** The intracellular ROS levels of NC group.


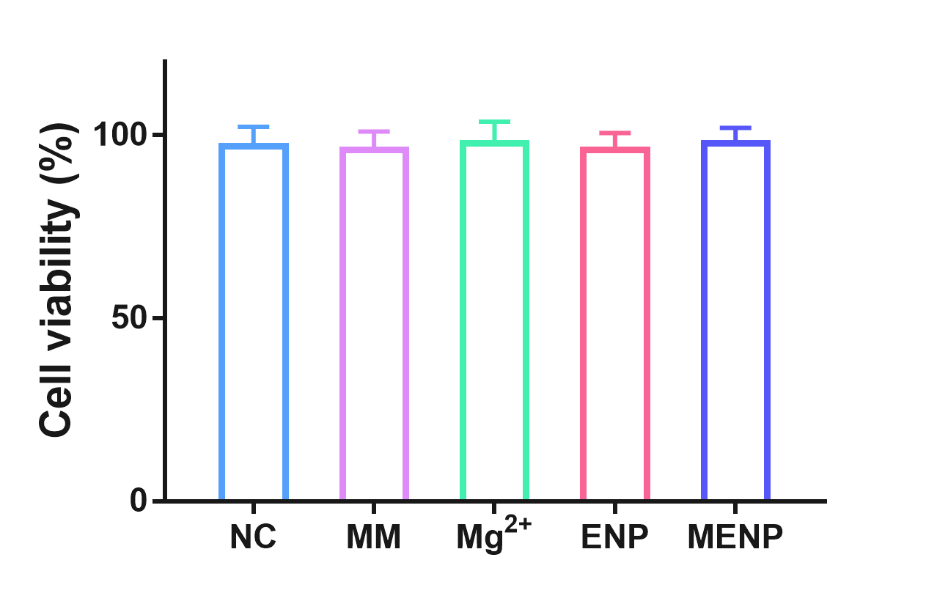


**Figure S5.** The viability of chondrocytes incubating different groups.


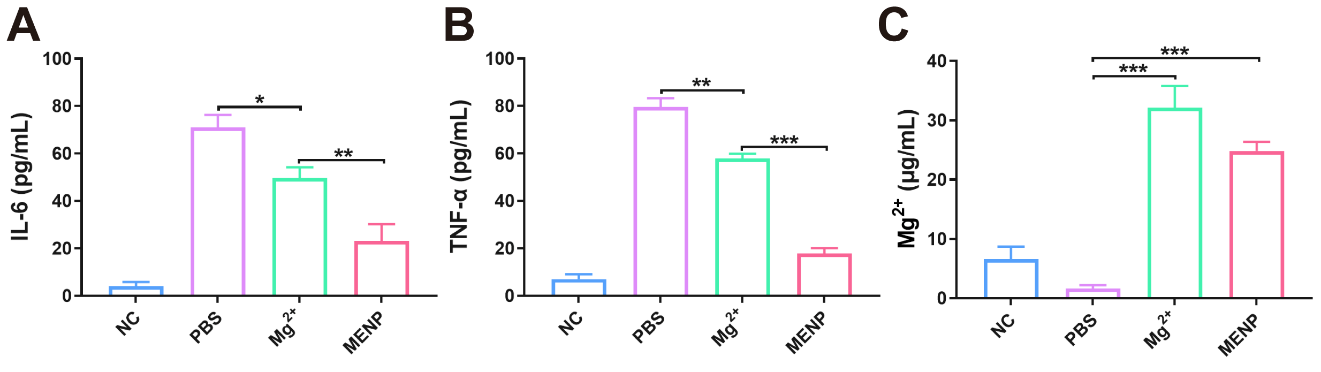


**Figure S6**. The levels of IL-6, TNF-α, and Mg^2+^ in the culture medium. ***P < 0.001, **P < 0.01, and *P < 0.05.


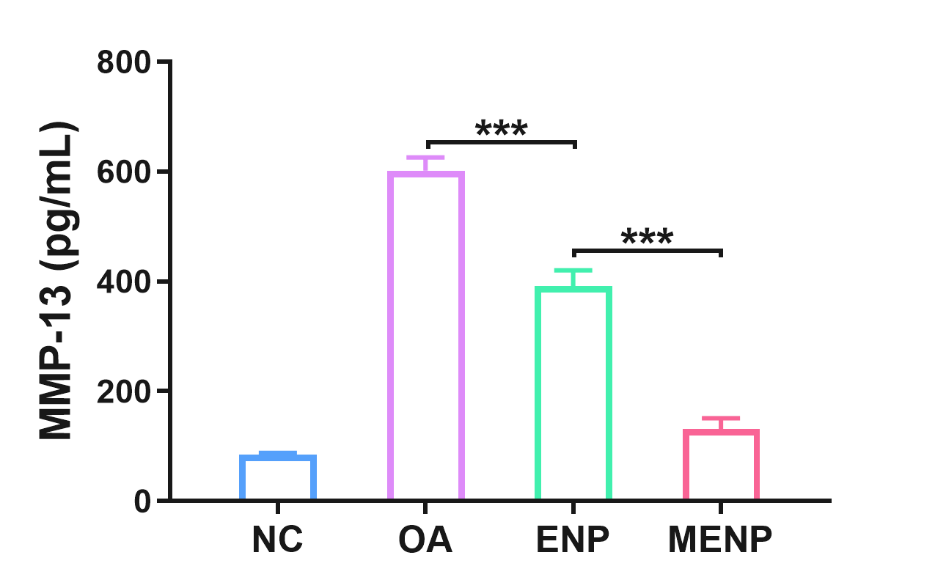


**Figure S7.** The expression of MMP-13. ***P < 0.001.


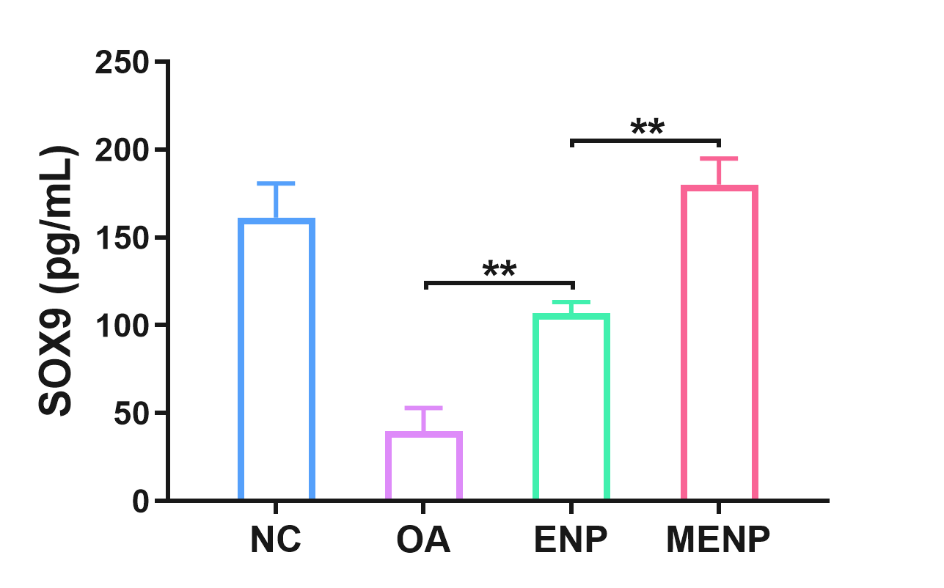


**Figure S8.** The expression of SOX9. **P < 0.01.


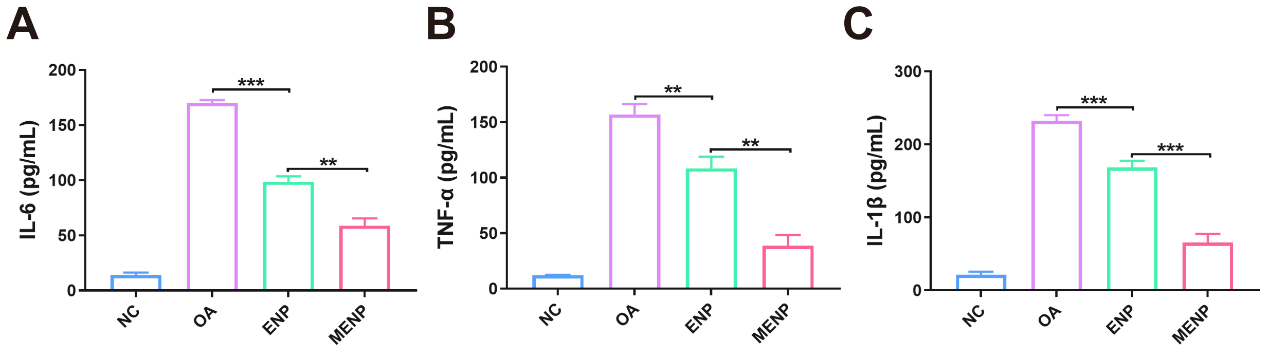


**Figure S9.** The expression of inflammatory cytokine (TNF-α, IL-1β, IL-6). ***P < 0.001, **P < 0.01.
